# Supplementary material for: A deficiency in SUMOylation activity disrupts multiple pathways leading to neural tube and heart defects in Xenopus embryos
Source: BMC Genomics. 2019 May 17;20:386. doi: 10.1186/s12864-019-5773-3 (PMC6525467; doi:10.1186/s12864-019-5773-3)
Supplement: Supplementary file 3 — Figure S2. Comparison of microarray data with measurement by qRT-PCR. The fold change between Gam1 and control embryos for the indicated gene as measured by microarray and qRT-PCR are compared with each pair of bars corresponding to early gastrula, late gastrula, and early neurula, respectively. Asterisks indicate samples in which the RNA level was too low to measure by qRT-PCR. (DOCX 157 kb) [file 12864_2019_5773_MOESM3_ESM.docx]

Table S3. Biological Processes Associated with Transcription Factors Identified in Network Building

**(Early Gastula)** Transcription factors highly associated with differentially expressed genes.

| Transcription Factor | Gene Ontology Processes | Number of Genes |
| --- | --- | --- |
| SP1 | camera‑type eye development (31.2%), eye development (31.2%), sensory organ development (31.2%), embryo development (37.5%), positive regulation of nucleobase, nucleoside, nucleotide and nucleic acid metabolic process (37.5%) | 15 |
| HNF4‑alpha | positive regulation of dendrite morphogenesis (13.3%), negative regulation of signaling pathway (26.7%), negative regulation of cellular process (53.3%), negative regulation of microtubule polymerization or depolymerization (13.3%), negative regulation of cell communication (26.7%) | 14 |
| c‑Myc | cell cycle process (42.9%), negative regulation of transcription, DNA‑dependent (35.7%), negative regulation of RNA metabolic process (35.7%), cell cycle (42.9%), detection of mechanical stimulus involved in sensory perception of sound (14.3%) | 13 |
| p53 | negative regulation of transcription from RNA polymerase II promoter (40.0%), negative regulation of transcription, DNA‑dependent (40.0%), negative regulation of RNA metabolic process (40.0%), negative regulation of transforming growth factor beta receptor signaling pathway (20.0%), cellular monovalent inorganic cation homeostasis (20.0%) | 9 |
| Oct‑3/4 | cell fate specification (37.5%), positive regulation of neurogenesis (37.5%), regulation of multicellular organismal development (62.5%), positive regulation of dendrite morphogenesis (25.0%), positive regulation of cell development (37.5%) | 7 |
| STAT3 | generation of neurons (71.4%), neurogenesis (71.4%), negative regulation of transcription from RNA polymerase II promoter (57.1%), negative regulation of transcription, DNA‑dependent (57.1%), negative regulation of RNA metabolic process (57.1%) | 6 |
| CREB1 | regulation of apoptosis (71.4%), regulation of programmed cell death (71.4%), regulation of cell death (71.4%), negative regulation of apoptosis (57.1%), negative regulation of programmed cell death (57.1%) | 6 |
| YY1 | negative regulation of transcription from RNA polymerase II promoter (66.7%), negative regulation of transcription, DNA‑dependent (66.7%), negative regulation of RNA metabolic process (66.7%), negative regulation of transcription (66.7%), negative regulation of gene expression (66.7%) | 5 |
| HNF1‑alpha | carbohydrate homeostasis (40.0%), glucose homeostasis (40.0%), negative regulation of chemokine production (20.0%), response to glucose stimulus (40.0%), response to hexose stimulus (40.0%) | 4 |
| Sry | positive regulation of transcription from RNA polymerase II promoter (80.0%), positive regulation of transcription, DNA‑dependent (80.0%), positive regulation of RNA metabolic process (80.0%), cell fate commitment (60.0%), regulation of transcription, DNA‑dependent (100.0%) | 4 |
| FKHR | regulation of molecular function (100.0%), regulation of MAPKKK cascade (60.0%), negative regulation of MAPKKK cascade (40.0%), negative regulation of biological process (100.0%), regulation of apoptosis (80.0%) | 4 |
| SRF | positive regulation of cell projection organization (60.0%), positive regulation of filopodium assembly (40.0%), regulation of filopodium assembly (40.0%), regulation of protein complex assembly (60.0%), positive regulation of dendrite morphogenesis (40.0%) | 4 |
| NANOG | somatic stem cell maintenance (40.0%), stem cell maintenance (40.0%), stem cell development (40.0%), negative regulation of transcription from RNA polymerase II promoter (60.0%), stem cell differentiation (40.0%) | 4 |
| E2F1 | response to growth factor stimulus (40.0%), nerve growth factor production (20.0%), DNA replication involved in S phase (20.0%), neurotrophin production (20.0%), zygotic determination of anterior/posterior axis, embryo (20.0%) | 4 |
| ETS1 | regulation of multicellular organismal development (80.0%), negative regulation of cellular process (100.0%), negative regulation of biological process (100.0%), regulation of BMP signaling pathway (40.0%), regulation of developmental process (80.0%) | 4 |
| EGR1 | learning or memory (60.0%), cognition (60.0%), cell differentiation (100.0%), cellular developmental process (100.0%), behavior (60.0%) | 4 |
| HIF1A | elastin metabolic process (20.0%), epithelial tube morphogenesis (40.0%), regulation of transcription from RNA polymerase II promoter (60.0%), positive regulation of macromolecule biosynthetic process (60.0%), connective tissue replacement involved in inflammatory response wound healing (20.0%) | 4 |
| AP‑2 | cellular lipid catabolic process (50.0%), neuropore closure (25.0%), anterior neuropore closure (25.0%), diacylglycerol catabolic process (25.0%), lipid catabolic process (50.0%) | 3 |
| ATF‑6 alpha | retina development in camera‑type eye (50.0%), positive regulation of gene‑specific transcription involved in unfolded protein response (25.0%), positive regulation of transcription, DNA‑dependent (75.0%), positive regulation of RNA metabolic process (75.0%), positive regulation of transcription (75.0%) | 3 |
| RelA (p65 NF‑kB subunit) | negative regulation of microtubule polymerization or depolymerization (50.0%), regulation of microtubule polymerization or depolymerization (50.0%), regulation of microtubule cytoskeleton organization (50.0%), regulation of microtubule‑based process (50.0%), negative regulation of cytoskeleton organization (50.0%) | 3 |
| C/EBPalpha | response to chemical stimulus (100.0%), response to vitamin B2 (25.0%), glucocorticoid mediated signaling pathway (25.0%), diacylglycerol catabolic process (25.0%), regulation of developmental process (75.0%) | 3 |
| ER81 | response to growth factor stimulus (50.0%), cell differentiation (100.0%), cellular developmental process (100.0%), positive regulation of transcription (75.0%), response to mechanical stimulus (50.0%) | 3 |
| HSF1 | snRNP protein import into nucleus (25.0%), rRNA modification (25.0%), embryonic process involved in female pregnancy (25.0%), regulation of microtubule depolymerization (25.0%), negative regulation of microtubule depolymerization (25.0%) | 3 |
| ESR1 (nuclear) | ovarian follicle development (50.0%), female gonad development (50.0%), development of primary female sexual characteristics (50.0%), ovulation cycle process (50.0%), female sex differentiation (50.0%) | 3 |
| USF2 | cellular lipid catabolic process (50.0%), lipid metabolic process (75.0%), positive regulation of transcription from RNA polymerase II promoter by carbon catabolites (25.0%), positive regulation of transcription by carbon catabolites (25.0%), regulation of transcription from RNA polymerase II promoter by carbon catabolites (25.0%) | 3 |
| AP‑1 | cellular response to reactive oxygen species (50.0%), cellular response to oxidative stress (50.0%), response to reactive oxygen species (50.0%), nerve growth factor production (25.0%), neurotrophin production (25.0%) | 3 |
| Androgen receptor | steroid hormone mediated signaling pathway (50.0%), chordate embryonic development (75.0%), embryo development ending in birth or egg hatching (75.0%), male somatic sex determination (25.0%), activation of prostate induction by androgen receptor signaling pathway (25.0%) | 3 |
| MYOD | myoblast cell fate determination (25.0%), positive regulation of skeletal muscle tissue regeneration (25.0%), regulation of skeletal muscle tissue regeneration (25.0%), muscle organ development (50.0%), myoblast cell fate commitment (25.0%) | 3 |
| FOXO3A | initiation of primordial ovarian follicle growth (25.0%), cellular response to chemical stimulus (75.0%), cellular response to stress (75.0%), cellular response to insulin stimulus (50.0%), cellular response to peptide hormone stimulus (50.0%) | 3 |
| AP‑2A | regulation of ossification (50.0%), nerve growth factor production (25.0%), detection of mechanical stimulus involved in equilibrioception (25.0%), neurotrophin production (25.0%), zygotic determination of anterior/posterior axis, embryo (25.0%) | 3 |

**(Early Gastula)** Transcription factors highly associated with unaffected genes.

| Transcription Factor | Gene Ontology Processes | Number of Genes |
| --- | --- | --- |
| HNF4‑alpha | cellular metabolic process (62.6%), metabolic process (68.7%), cellular process (81.2%), primary metabolic process (59.8%), cellular macromolecule metabolic process (47.0%) | 1372 |
| SP1 | cellular process (90.6%), cellular metabolic process (68.1%), metabolic process (75.0%), primary metabolic process (67.2%), developmental process (46.2%) | 1082 |
| c‑Myc | cellular metabolic process (69.4%), cellular process (88.3%), primary metabolic process (67.9%), metabolic process (73.9%), cellular macromolecule metabolic process (52.9%) | 1033 |
| p53 | cellular process (90.7%), negative regulation of cellular process (35.9%), negative regulation of biological process (36.5%), positive regulation of cellular process (36.7%), regulation of cell cycle (17.0%) | 486 |
| ESR1 (nuclear) | cellular process (82.5%), positive regulation of biological process (33.5%), cellular metabolic process (60.0%), primary metabolic process (59.6%), response to organic substance (21.9%) | 479 |
| CREB1 | cellular process (89.5%), response to organic substance (28.4%), negative regulation of cellular process (34.4%), negative regulation of biological process (35.9%), cellular metabolic process (65.6%) | 467 |
| AP‑1 | response to organic substance (32.7%), cellular process (90.4%), response to chemical stimulus (42.8%), response to endogenous stimulus (23.7%), response to stimulus (54.5%) | 378 |
| NF‑Y | cellular response to stimulus (28.6%), cell cycle (23.1%), cellular process (87.0%), developmental process (46.8%), primary metabolic process (66.6%) | 310 |
| E2F1 | DNA metabolic process (23.3%), cell cycle (26.8%), DNA replication (14.5%), cellular macromolecule metabolic process (62.5%), cellular metabolic process (73.2%) | 342 |
| NF‑kB | response to organic substance (34.5%), regulation of programmed cell death (29.1%), regulation of cell death (29.1%), regulation of apoptosis (28.4%), positive regulation of biological process (44.7%) | 314 |
| AP‑2 | response to organic substance (31.5%), response to chemical stimulus (42.0%), primary metabolic process (70.8%), cellular process (89.2%), cellular metabolic process (69.2%) | 306 |
| YY1 | translational elongation (15.7%), cellular metabolic process (76.3%), cellular process (91.7%), metabolic process (79.7%), cellular biosynthetic process (50.3%) | 303 |
| EGR1 | anatomical structure development (50.0%), developmental process (54.7%), cellular process (91.9%), response to organic substance (33.4%), multicellular organismal development (50.0%) | 300 |
| GATA‑1 | positive regulation of biological process (45.5%), positive regulation of cellular process (43.0%), developmental process (52.7%), negative regulation of cellular process (38.4%), negative regulation of biological process (40.1%) | 283 |
| Elk‑1 | cellular protein metabolic process (37.9%), cellular metabolic process (69.2%), cellular macromolecule metabolic process (57.9%), primary metabolic process (68.7%), protein metabolic process (40.2%) | 214 |
| ETS1 | macromolecule metabolic process (68.4%), positive regulation of macromolecule metabolic process (33.6%), positive regulation of cellular metabolic process (34.0%), positive regulation of metabolic process (34.8%), primary metabolic process (76.1%) | 250 |
| Androgen receptor | cellular process (90.6%), positive regulation of macromolecule metabolic process (26.4%), positive regulation of biological process (39.4%), regulation of cell cycle (17.3%), positive regulation of cellular process (36.6%) | 258 |
| HIF1A | positive regulation of cellular process (47.2%), positive regulation of biological process (48.1%), cellular process (93.1%), positive regulation of metabolic process (34.3%), positive regulation of cellular metabolic process (33.5%) | 236 |
| C/EBPbeta | response to organic substance (40.9%), response to chemical stimulus (48.5%), positive regulation of biological process (48.9%), positive regulation of cellular process (46.0%), developmental process (55.7%) | 235 |
| Oct‑3/4 | negative regulation of gene expression (30.0%), regulation of transcription (52.7%), regulation of cellular macromolecule biosynthetic process (54.9%), regulation of gene expression (55.7%), regulation of transcription, DNA‑dependent (44.3%) | 236 |
| c‑Jun | response to organic substance (36.1%), developmental process (57.0%), positive regulation of biological process (48.3%), positive regulation of cellular process (45.7%), cellular process (93.0%) | 233 |
| E2F4 | cell cycle (33.6%), DNA metabolic process (26.5%), DNA replication (18.1%), regulation of cell cycle (26.1%), cellular process (95.1%) | 229 |
| RelA (p65 NF‑kB subunit) | positive regulation of biological process (51.8%), positive regulation of cellular process (48.2%), response to organic substance (36.0%), response to chemical stimulus (45.0%), negative regulation of biological process (42.8%) | 224 |
| SP3 | developmental process (60.5%), anatomical structure development (55.6%), multicellular organismal development (55.6%), organ development (43.9%), response to organic substance (36.6%) | 206 |
| GCR‑alpha | cellular process (93.2%), positive regulation of biological process (46.3%), negative regulation of cellular process (40.0%), response to organic substance (32.2%), negative regulation of biological process (41.0%) | 205 |
| HNF6 | cellular metabolic process (73.7%), metabolic process (79.0%), positive regulation of macromolecule metabolic process (29.8%), positive regulation of cellular metabolic process (30.2%), primary metabolic process (70.7%) | 205 |
| Oct‑1 | positive regulation of biological process (48.7%), developmental process (56.9%), negative regulation of cellular process (43.1%), positive regulation of cellular process (45.7%), negative regulation of biological process (44.2%) | 202 |
| AP‑2A | cellular process (94.5%), developmental process (57.7%), negative regulation of cellular process (42.8%), positive regulation of cellular process (44.8%), positive regulation of biological process (46.8%) | 200 |
| AHR | positive regulation of cellular process (46.6%), positive regulation of biological process (47.6%), positive regulation of gene expression (27.5%), positive regulation of metabolic process (34.4%), positive regulation of nitrogen compound metabolic process (28.0%) | 192 |
| HNF1‑alpha | positive regulation of gene expression (26.7%), positive regulation of transcription from RNA polymerase II promoter (22.0%), positive regulation of transcription (25.7%), cellular metabolic process (73.3%), positive regulation of transcription, DNA‑dependent (23.0%) | 192 |

**(Late Gastula)** Transcription factors highly associated with differentially expressed genes.

| Transcription Factor | Gene Ontology Processes | Number of Genes |
| --- | --- | --- |
| HNF4‑alpha | nucleic acid metabolic process (48.5%), nucleobase, nucleoside, nucleotide and nucleic acid metabolic process (52.5%), nitrogen compound metabolic process (54.5%), cellular metabolic process (72.3%), cellular nitrogen compound metabolic process (53.5%) | 104 |
| SP1 | organ development (54.5%), system development (57.6%), multicellular organismal development (61.6%), anatomical structure development (59.6%), developmental process (61.6%) | 101 |
| c‑Myc | cellular macromolecule biosynthetic process (50.6%), macromolecule biosynthetic process (50.6%), regulation of nucleobase, nucleoside, nucleotide and nucleic acid metabolic process (52.8%), negative regulation of transcription (25.8%), negative regulation of transcription, DNA‑dependent (23.6%) | 92 |
| ESR1 (nuclear) | regulation of transcription from RNA polymerase II promoter (42.5%), positive regulation of transcription, DNA‑dependent (37.0%), positive regulation of RNA metabolic process (37.0%), positive regulation of transcription (38.4%), positive regulation of nucleobase, nucleoside, nucleotide and nucleic acid metabolic process (39.7%) | 75 |
| p53 | regulation of transcription from RNA polymerase II promoter (39.1%), positive regulation of gene expression (37.5%), positive regulation of transcription (35.9%), positive regulation of nucleobase, nucleoside, nucleotide and nucleic acid metabolic process (37.5%), positive regulation of transcription, DNA‑dependent (32.8%) | 67 |
| E2F1 | nucleic acid metabolic process (69.0%), positive regulation of macromolecule metabolic process (50.0%), cellular macromolecule metabolic process (82.8%), positive regulation of cellular metabolic process (50.0%), negative regulation of transcription (37.9%) | 60 |
| NF‑Y | organ development (61.4%), regulation of transcription from RNA polymerase II promoter (45.6%), multicellular organismal development (70.2%), positive regulation of transcription, DNA‑dependent (36.8%), system development (63.2%) | 59 |
| Androgen receptor | regulation of transcription from RNA polymerase II promoter (48.2%), positive regulation of transcription, DNA‑dependent (42.9%), positive regulation of gene expression (46.4%), positive regulation of RNA metabolic process (42.9%), positive regulation of transcription (44.6%) | 58 |
| EGR1 | regulation of transcription from RNA polymerase II promoter (51.0%), positive regulation of transcription, DNA‑dependent (43.1%), positive regulation of RNA metabolic process (43.1%), positive regulation of nucleobase, nucleoside, nucleotide and nucleic acid metabolic process (47.1%), positive regulation of macromolecule metabolic process (52.9%) | 54 |
| c‑Jun | regulation of transcription from RNA polymerase II promoter (49.0%), positive regulation of transcription, DNA‑dependent (43.1%), positive regulation of RNA metabolic process (43.1%), positive regulation of transcription from RNA polymerase II promoter (39.2%), positive regulation of gene expression (45.1%) | 53 |
| ETS1 | positive regulation of nucleobase, nucleoside, nucleotide and nucleic acid metabolic process (52.9%), positive regulation of macromolecule metabolic process (58.8%), positive regulation of nitrogen compound metabolic process (52.9%), positive regulation of gene expression (51.0%), positive regulation of macromolecule biosynthetic process (52.9%) | 53 |
| GCR‑alpha | positive regulation of transcription, DNA‑dependent (39.2%), positive regulation of RNA metabolic process (39.2%), positive regulation of transcription (41.2%), regulation of transcription from RNA polymerase II promoter (43.1%), positive regulation of gene expression (41.2%) | 53 |
| YY1 | regulation of transcription from RNA polymerase II promoter (50.0%), negative regulation of transcription, DNA‑dependent (38.0%), positive regulation of metabolic process (54.0%), negative regulation of RNA metabolic process (38.0%), positive regulation of macromolecule metabolic process (52.0%) | 52 |
| Oct‑3/4 | regulation of transcription, DNA‑dependent (69.4%), regulation of RNA metabolic process (69.4%), regulation of transcription from RNA polymerase II promoter (55.1%), positive regulation of transcription, DNA‑dependent (49.0%), positive regulation of RNA metabolic process (49.0%) | 51 |
| RelA (p65 NF‑kB subunit) | regulation of transcription from RNA polymerase II promoter (49.0%), positive regulation of transcription, DNA‑dependent (42.9%), positive regulation of RNA metabolic process (42.9%), negative regulation of transcription, DNA‑dependent (38.8%), negative regulation of RNA metabolic process (38.8%) | 51 |
| Oct‑1 | regulation of transcription from RNA polymerase II promoter (56.2%), positive regulation of transcription, DNA‑dependent (50.0%), positive regulation of RNA metabolic process (50.0%), positive regulation of transcription from RNA polymerase II promoter (45.8%), positive regulation of transcription (50.0%) | 50 |
| SRF | regulation of transcription from RNA polymerase II promoter (58.3%), positive regulation of transcription, DNA‑dependent (50.0%), positive regulation of RNA metabolic process (50.0%), regulation of transcription, DNA‑dependent (68.8%), regulation of RNA metabolic process (68.8%) | 50 |
| HIF1A | regulation of transcription from RNA polymerase II promoter (52.1%), positive regulation of transcription, DNA‑dependent (45.8%), positive regulation of RNA metabolic process (45.8%), positive regulation of transcription from RNA polymerase II promoter (41.7%), positive regulation of macromolecule biosynthetic process (50.0%) | 50 |
| MYOD | regulation of transcription from RNA polymerase II promoter (57.4%), positive regulation of transcription, DNA‑dependent (51.1%), positive regulation of RNA metabolic process (51.1%), positive regulation of transcription from RNA polymerase II promoter (46.8%), regulation of transcription, DNA‑dependent (68.1%) | 49 |
| C/EBPbeta | regulation of transcription from RNA polymerase II promoter (51.1%), positive regulation of transcription, DNA‑dependent (44.7%), positive regulation of RNA metabolic process (44.7%), positive regulation of transcription from RNA polymerase II promoter (40.4%), negative regulation of gene expression (42.6%) | 49 |
| Bcl‑6 | negative regulation of transcription (50.0%), negative regulation of gene expression (50.0%), negative regulation of nucleobase, nucleoside, nucleotide and nucleic acid metabolic process (50.0%), negative regulation of cellular macromolecule biosynthetic process (50.0%), negative regulation of nitrogen compound metabolic process (50.0%) | 48 |
| TCF7L2 (TCF4) | negative regulation of transcription (50.0%), regulation of transcription, DNA‑dependent (69.6%), regulation of RNA metabolic process (69.6%), negative regulation of gene expression (50.0%), negative regulation of nucleobase, nucleoside, nucleotide and nucleic acid metabolic process (50.0%) | 48 |
| HSF1 | regulation of transcription from RNA polymerase II promoter (58.7%), regulation of transcription, DNA‑dependent (71.7%), regulation of RNA metabolic process (71.7%), positive regulation of transcription, DNA‑dependent (50.0%), positive regulation of RNA metabolic process (50.0%) | 48 |
| HNF6 | regulation of transcription from RNA polymerase II promoter (54.3%), negative regulation of transcription (45.7%), regulation of transcription, DNA‑dependent (65.2%), regulation of RNA metabolic process (65.2%), positive regulation of transcription, DNA‑dependent (45.7%) | 48 |
| E2F4 | nucleic acid metabolic process (75.6%), regulation of transcription from RNA polymerase II promoter (48.9%), nucleobase, nucleoside, nucleotide and nucleic acid metabolic process (75.6%), positive regulation of transcription, DNA‑dependent (40.0%), positive regulation of RNA metabolic process (40.0%) | 47 |
| SMAD3 | positive regulation of transcription, DNA‑dependent (56.8%), positive regulation of RNA metabolic process (56.8%), regulation of transcription from RNA polymerase II promoter (61.4%), positive regulation of transcription (56.8%), regulation of transcription, DNA‑dependent (72.7%) | 47 |
| SOX4 | regulation of transcription from RNA polymerase II promoter (55.6%), regulation of transcription, DNA‑dependent (66.7%), regulation of RNA metabolic process (66.7%), negative regulation of transcription, DNA‑dependent (42.2%), negative regulation of RNA metabolic process (42.2%) | 47 |
| STAT1 | regulation of transcription from RNA polymerase II promoter (47.7%), positive regulation of transcription, DNA‑dependent (40.9%), positive regulation of nucleobase, nucleoside, nucleotide and nucleic acid metabolic process (45.5%), positive regulation of RNA metabolic process (40.9%), positive regulation of nitrogen compound metabolic process (45.5%) | 46 |
| c‑Myb | regulation of transcription from RNA polymerase II promoter (52.3%), positive regulation of transcription, DNA‑dependent (45.5%), positive regulation of RNA metabolic process (45.5%), regulation of transcription, DNA‑dependent (63.6%), regulation of macromolecule metabolic process (79.5%) | 46 |
| SREBP1 (nuclear) | regulation of transcription from RNA polymerase II promoter (48.8%), positive regulation of transcription, DNA‑dependent (41.9%), positive regulation of RNA metabolic process (41.9%), negative regulation of transcription, DNA‑dependent (37.2%), negative regulation of RNA metabolic process (37.2%) | 46 |

**(Later Gastula)** Transcription factors highly associated with unaffected genes.

| Transcription Factor | Gene Ontology Processes | Number of Genes |
| --- | --- | --- |
| HNF4‑alpha | cellular metabolic process (62.3%), metabolic process (68.5%), cellular process (81.4%), primary metabolic process (59.5%), cellular macromolecule metabolic process (46.7%) | 1335 |
| SP1 | cellular process (90.8%), cellular metabolic process (68.3%), metabolic process (75.2%), primary metabolic process (67.2%), developmental process (45.9%) | 1053 |
| c‑Myc | cellular process (88.4%), cellular metabolic process (69.0%), primary metabolic process (67.5%), metabolic process (73.5%), cellular macromolecule metabolic process (52.6%) | 1002 |
| p53 | cellular process (90.8%), positive regulation of cellular process (38.8%), positive regulation of biological process (40.1%), negative regulation of cellular process (35.3%), regulation of cell cycle (17.4%) | 461 |
| ESR1 (nuclear) | cellular process (82.8%), cellular metabolic process (61.1%), primary metabolic process (60.7%), positive regulation of biological process (33.3%), metabolic process (66.5%) | 463 |
| CREB1 | cellular process (89.6%), response to organic substance (27.9%), negative regulation of cellular process (34.4%), negative regulation of biological process (36.1%), cellular metabolic process (65.4%) | 452 |
| AP‑1 | response to organic substance (32.5%), cellular process (90.4%), response to chemical stimulus (42.9%), response to stimulus (54.9%), response to endogenous stimulus (23.5%) | 370 |
| E2F1 | DNA metabolic process (22.9%), cellular process (92.2%), DNA replication (14.5%), cell cycle (26.5%), cellular metabolic process (72.9%) | 335 |
| NF‑Y | cell cycle (23.7%), cellular response to stimulus (28.1%), cellular process (87.3%), regulation of cell cycle (17.4%), cell cycle process (18.7%) | 301 |
| NF‑kB | response to organic substance (35.2%), regulation of programmed cell death (29.6%), regulation of cell death (29.6%), regulation of apoptosis (28.9%), positive regulation of biological process (45.5%) | 302 |
| AP‑2 | cellular process (89.7%), primary metabolic process (70.0%), cellular metabolic process (69.3%), positive regulation of biological process (40.7%), metabolic process (75.3%) | 300 |
| EGR1 | cellular process (92.8%), developmental process (55.2%), anatomical structure development (50.3%), response to organic substance (33.4%), multicellular organismal development (50.3%) | 293 |
| YY1 | translational elongation (15.6%), cellular metabolic process (76.0%), cellular process (91.7%), translation (17.4%), cellular biosynthetic process (50.0%) | 290 |
| GATA‑1 | positive regulation of biological process (44.1%), positive regulation of cellular process (41.5%), developmental process (50.7%), cellular process (89.0%), negative regulation of cellular process (37.1%) | 276 |
| Elk‑1 | cellular metabolic process (70.1%), cellular protein metabolic process (38.4%), cellular macromolecule metabolic process (58.8%), primary metabolic process (69.7%), cellular process (88.2%) | 211 |
| ETS1 | macromolecule metabolic process (69.0%), primary metabolic process (76.7%), positive regulation of cellular process (44.5%), positive regulation of macromolecule metabolic process (32.2%), positive regulation of metabolic process (33.5%) | 248 |
| Androgen receptor | cellular process (90.5%), positive regulation of cellular process (38.9%), positive regulation of biological process (40.9%), developmental process (48.0%), positive regulation of macromolecule metabolic process (26.6%) | 255 |
| HIF1A | positive regulation of cellular process (46.8%), cellular process (93.5%), positive regulation of biological process (48.1%), developmental process (55.8%), positive regulation of metabolic process (33.8%) | 233 |
| C/EBPbeta | response to organic substance (39.1%), response to chemical stimulus (47.4%), positive regulation of biological process (47.8%), positive regulation of cellular process (44.8%), cellular process (91.7%) | 230 |
| E2F4 | cell cycle (34.7%), DNA replication (18.2%), DNA metabolic process (26.2%), regulation of cell cycle (25.8%), cellular process (95.6%) | 228 |
| Oct‑3/4 | regulation of cellular macromolecule biosynthetic process (53.1%), regulation of transcription (50.9%), negative regulation of gene expression (28.1%), regulation of transcription, DNA‑dependent (43.0%), regulation of RNA metabolic process (43.4%) | 227 |
| c‑Jun | developmental process (57.7%), positive regulation of biological process (48.2%), cellular process (93.2%), positive regulation of cellular process (45.5%), anatomical structure development (50.9%) | 222 |
| RelA (p65 NF‑kB subunit) | positive regulation of biological process (51.9%), positive regulation of cellular process (48.6%), response to organic substance (35.5%), negative regulation of biological process (43.5%), regulation of cell death (29.4%) | 216 |
| SP3 | developmental process (60.4%), anatomical structure development (55.4%), multicellular organismal development (55.4%), organ development (43.6%), response to organic substance (36.6%) | 203 |
| SRF | developmental process (57.1%), anatomical structure development (51.6%), anatomical structure morphogenesis (34.8%), positive regulation of cellular process (44.6%), positive regulation of macromolecule metabolic process (32.1%) | 188 |
| AP‑2A | cellular process (94.4%), developmental process (57.6%), negative regulation of cellular process (43.9%), negative regulation of biological process (44.9%), positive regulation of cellular process (44.4%) | 197 |
| HNF6 | cellular metabolic process (73.1%), metabolic process (78.2%), positive regulation of macromolecule metabolic process (29.4%), positive regulation of cellular metabolic process (29.9%), positive regulation of metabolic process (29.9%) | 197 |
| Oct‑1 | positive regulation of biological process (49.5%), positive regulation of cellular process (46.3%), developmental process (56.4%), negative regulation of cellular process (41.5%), primary metabolic process (74.5%) | 195 |
| GCR‑alpha | cellular process (93.8%), positive regulation of biological process (45.6%), negative regulation of cellular process (40.5%), negative regulation of biological process (41.5%), response to organic substance (32.3%) | 195 |
| AHR | positive regulation of cellular process (46.8%), positive regulation of biological process (47.8%), positive regulation of metabolic process (34.4%), positive regulation of gene expression (26.9%), positive regulation of macromolecule metabolic process (32.3%) | 189 |

**(Early Neurula)** Transcription factors highly associated with differentially expressed genes.

| Transcription Factor | Gene Ontology Processes | Number of Genes |
| --- | --- | --- |
| SP1 | cellular process (95.0%), primary metabolic process (70.8%), developmental process (50.0%), metabolic process (76.7%), cellular metabolic process (69.2%) | 119 |
| HNF4‑alpha | cellular process (85.7%), cellular metabolic process (64.3%), primary metabolic process (62.5%), metabolic process (68.8%), cellular macromolecule metabolic process (46.4%) | 114 |
| c‑Myc | cellular process (94.4%), cellular metabolic process (76.6%), primary metabolic process (76.6%), metabolic process (80.4%), cellular macromolecule metabolic process (60.7%) | 106 |
| p53 | cellular process (94.9%), regulation of biological process (83.3%), regulation of cellular process (80.8%), cellular macromolecule metabolic process (65.4%), biological regulation (84.6%) | 77 |
| ESR1 (nuclear) | cellular process (95.7%), cellular metabolic process (73.9%), biological regulation (81.2%), anatomical structure development (47.8%), primary metabolic process (72.5%) | 69 |
| C/EBPbeta | response to stress (49.0%), cellular response to stimulus (39.2%), cellular process (96.1%), regulation of apoptosis (33.3%), positive regulation of transcription, DNA‑dependent (27.5%) | 50 |
| NF‑kB | apoptosis (28.3%), programmed cell death (28.3%), response to reactive oxygen species (15.2%), positive regulation of transcription, DNA‑dependent (26.1%), positive regulation of RNA metabolic process (26.1%) | 45 |
| STAT1 | positive regulation of biological process (53.8%), positive regulation of nucleobase, nucleoside, nucleotide and nucleic acid metabolic process (33.3%), positive regulation of macromolecule metabolic process (38.5%), positive regulation of cellular process (51.3%), positive regulation of nitrogen compound metabolic process (33.3%) | 38 |
| Androgen receptor | cellular process (100.0%), cell death (31.6%), death (31.6%), developmental process (60.5%), anatomical structure development (55.3%) | 37 |
| CREB1 | cellular response to chemical stimulus (23.7%), embryonic organ development (15.8%), primary metabolic process (71.1%), regulation of apoptosis (26.3%), cellular process (89.5%) | 37 |
| HSF1 | regulation of macromolecule metabolic process (70.3%), regulation of gene expression (64.9%), cellular macromolecule metabolic process (78.4%), embryo development (37.8%), gene expression (62.2%) | 36 |
| MYOD | positive regulation of transcription, DNA‑dependent (45.9%), positive regulation of RNA metabolic process (45.9%), regulation of macromolecule metabolic process (78.4%), positive regulation of nucleobase, nucleoside, nucleotide and nucleic acid metabolic process (48.6%), positive regulation of nitrogen compound metabolic process (48.6%) | 36 |
| SRF | regulation of transcription, DNA‑dependent (62.2%), regulation of transcription from RNA polymerase II promoter (48.6%), regulation of RNA metabolic process (62.2%), regulation of transcription (64.9%), positive regulation of transcription, DNA‑dependent (37.8%) | 36 |
| EGR1 | developmental process (67.6%), multicellular organismal development (59.5%), cellular macromolecule metabolic process (70.3%), positive regulation of macromolecule metabolic process (37.8%), cell differentiation (45.9%) | 36 |
| E2F1 | cellular metabolic process (91.9%), cellular macromolecule metabolic process (81.1%), primary metabolic process (89.2%), macromolecule metabolic process (81.1%), metabolic process (91.9%) | 36 |
| AP‑1 | response to chemical stimulus (54.3%), cellular response to chemical stimulus (31.4%), embryo development (31.4%), cellular response to stimulus (37.1%), regulation of monocyte differentiation (8.6%) | 35 |
| RelA (p65 NF‑kB subunit) | cell differentiation (51.4%), cellular developmental process (51.4%), developmental process (65.7%), anatomical structure development (60.0%), positive regulation of DNA replication (14.3%) | 34 |
| C/EBPalpha | regulation of macromolecule metabolic process (72.7%), regulation of metabolic process (78.8%), regulation of transcription, DNA‑dependent (54.5%), regulation of RNA metabolic process (54.5%), positive regulation of transcription, DNA‑dependent (36.4%) | 32 |
| p21 | regulation of transcription (78.8%), regulation of transcription, DNA‑dependent (69.7%), regulation of RNA metabolic process (69.7%), regulation of cellular macromolecule biosynthetic process (78.8%), regulation of macromolecule biosynthetic process (78.8%) | 32 |
| ATF‑2 | macromolecule metabolic process (84.4%), response to hydrogen peroxide (21.9%), positive regulation of apoptosis (34.4%), positive regulation of programmed cell death (34.4%), cellular response to stress (37.5%) | 31 |
| YY1 | cellular metabolic process (93.3%), cellular biosynthetic process (70.0%), cellular macromolecule biosynthetic process (63.3%), biosynthetic process (70.0%), macromolecule biosynthetic process (63.3%) | 29 |
| HIF1A | response to oxygen levels (23.3%), response to hypoxia (20.0%), regulation of transforming growth factor‑beta production (10.0%), positive regulation of myeloid cell differentiation (13.3%), response to stress (46.7%) | 29 |
| p63 | cellular response to stress (36.7%), metabolic process (93.3%), macromolecule metabolic process (80.0%), cellular macromolecule metabolic process (73.3%), response to DNA damage stimulus (26.7%) | 29 |
| SP3 | developmental process (65.5%), cellular process (100.0%), anatomical structure development (58.6%), multicellular organismal development (58.6%), interspecies interaction between organisms (24.1%) | 28 |
| NF‑Y | cellular response to stimulus (44.8%), response to chemical stimulus (55.2%), response to organic substance (41.4%), response to stress (48.3%), protein oligomerization (20.7%) | 28 |
| AP‑4 | positive regulation of transcription, DNA‑dependent (41.4%), positive regulation of RNA metabolic process (41.4%), positive regulation of transcription from RNA polymerase II promoter (37.9%), positive regulation of transcription (41.4%), positive regulation by host of viral transcription (13.8%) | 28 |
| c‑Jun | response to oxidative stress (29.6%), cellular metabolic process (92.6%), response to hydrogen peroxide (22.2%), response to reactive oxygen species (22.2%), metabolic process (92.6%) | 27 |
| GATA‑1 | positive regulation of biological process (65.4%), positive regulation of cellular process (61.5%), regulation of metabolic process (73.1%), regulation of macromolecule metabolic process (65.4%), positive regulation of macromolecule metabolic process (42.3%) | 26 |
| STAT3 | regulation of transcription, DNA‑dependent (59.3%), regulation of RNA metabolic process (59.3%), regulation of transcription from RNA polymerase II promoter (44.4%), cellular macromolecule metabolic process (81.5%), regulation of transcription (63.0%) | 26 |
| Oct‑3/4 | embryo development (60.0%), embryonic morphogenesis (48.0%), organ development (72.0%), anatomical structure morphogenesis (64.0%), tissue development (56.0%) | 24 |

**(Early Neurula)** Transcription factors highly associated with unaffected genes.

| Transcription Factor | Gene Ontology Processes | Number of Genes |
| --- | --- | --- |
| HNF4‑alpha | cellular metabolic process (62.8%), metabolic process (69.0%), cellular process (81.3%), primary metabolic process (59.9%), cellular macromolecule metabolic process (47.3%) | 1313 |
| SP1 | cellular process (90.7%), cellular metabolic process (68.8%), metabolic process (75.6%), primary metabolic process (67.7%), developmental process (46.3%) | 1028 |
| c‑Myc | cellular metabolic process (69.1%), cellular process (87.9%), primary metabolic process (67.5%), metabolic process (73.7%), cellular macromolecule metabolic process (52.7%) | 987 |
| p53 | cellular process (90.8%), negative regulation of cellular process (35.2%), positive regulation of cellular process (37.8%), regulation of cell cycle (17.7%), positive regulation of biological process (38.9%) | 460 |
| ESR1 (nuclear) | cellular process (82.3%), cellular metabolic process (60.9%), positive regulation of biological process (33.8%), primary metabolic process (60.7%), response to organic substance (22.1%) | 455 |
| CREB1 | cellular process (89.5%), response to organic substance (28.1%), negative regulation of cellular process (34.5%), negative regulation of biological process (36.1%), cellular metabolic process (65.5%) | 451 |
| AP‑1 | response to organic substance (33.2%), response to chemical stimulus (43.8%), cellular process (90.3%), response to stimulus (55.7%), response to endogenous stimulus (24.1%) | 365 |
| E2F1 | DNA metabolic process (22.5%), cellular process (91.8%), cellular metabolic process (72.9%), cellular macromolecule metabolic process (62.0%), macromolecule metabolic process (64.7%) | 332 |
| NF‑Y | cell cycle (23.9%), cellular process (87.7%), regulation of cell cycle (17.6%), developmental process (47.2%), cell cycle process (18.6%) | 303 |
| NF‑kB | response to organic substance (34.3%), regulation of programmed cell death (28.6%), positive regulation of biological process (45.1%), regulation of cell death (28.6%), response to chemical stimulus (44.1%) | 298 |
| YY1 | translational elongation (16.2%), cellular metabolic process (77.0%), cellular process (91.4%), cellular biosynthetic process (50.9%), translation (17.5%) | 294 |
| AP‑2 | response to organic substance (31.2%), primary metabolic process (71.2%), response to chemical stimulus (42.1%), cellular metabolic process (70.2%), cellular process (89.0%) | 293 |
| EGR1 | anatomical structure development (50.5%), cellular process (92.3%), developmental process (54.4%), response to organic substance (33.7%), organ development (38.9%) | 289 |
| GATA‑1 | positive regulation of biological process (44.1%), positive regulation of cellular process (41.8%), developmental process (51.7%), negative regulation of cellular process (38.0%), negative regulation of biological process (39.5%) | 266 |
| Elk‑1 | cellular protein metabolic process (38.8%), cellular macromolecule metabolic process (59.7%), cellular metabolic process (69.9%), macromolecule metabolic process (61.7%), protein metabolic process (41.3%) | 206 |
| ETS1 | macromolecule metabolic process (68.6%), positive regulation of macromolecule metabolic process (33.9%), primary metabolic process (76.6%), positive regulation of metabolic process (35.1%), positive regulation of cellular metabolic process (34.3%) | 242 |
| Androgen receptor | cellular process (90.2%), positive regulation of macromolecule metabolic process (26.5%), positive regulation of biological process (39.6%), positive regulation of cellular process (37.1%), cellular metabolic process (66.1%) | 249 |
| Oct‑3/4 | negative regulation of gene expression (29.6%), regulation of transcription (52.4%), regulation of gene expression (55.4%), negative regulation of transcription (27.9%), regulation of cellular macromolecule biosynthetic process (54.1%) | 232 |
| c‑Jun | developmental process (59.3%), positive regulation of biological process (50.2%), organ development (43.4%), positive regulation of cellular process (47.1%), anatomical structure development (52.5%) | 224 |
| E2F4 | cell cycle (34.1%), DNA metabolic process (25.9%), regulation of cell cycle (25.9%), DNA replication (17.3%), cellular process (95.0%) | 223 |
| HIF1A | positive regulation of cellular process (48.4%), positive regulation of biological process (48.9%), positive regulation of metabolic process (35.6%), positive regulation of macromolecule metabolic process (33.8%), cellular process (93.6%) | 222 |
| C/EBPbeta | response to organic substance (41.0%), response to chemical stimulus (49.3%), positive regulation of biological process (49.3%), positive regulation of cellular process (46.5%), developmental process (56.7%) | 217 |
| RelA (p65 NF‑kB subunit) | positive regulation of biological process (51.4%), positive regulation of cellular process (48.1%), response to organic substance (36.3%), negative regulation of biological process (44.3%), negative regulation of cellular process (41.0%) | 214 |
| SP3 | anatomical structure development (55.6%), organ development (45.5%), developmental process (60.1%), response to organic substance (37.4%), multicellular organismal development (55.6%) | 199 |
| GCR‑alpha | cellular process (92.9%), response to organic substance (33.3%), positive regulation of biological process (45.5%), negative regulation of cellular process (39.9%), negative regulation of biological process (41.4%) | 198 |
| HNF6 | cellular metabolic process (73.7%), positive regulation of macromolecule metabolic process (30.8%), positive regulation of cellular metabolic process (31.3%), metabolic process (79.3%), positive regulation of metabolic process (31.3%) | 198 |
| AP‑2A | cellular process (94.9%), developmental process (57.9%), negative regulation of cellular process (43.6%), negative regulation of biological process (44.6%), cellular metabolic process (74.9%) | 194 |
| Oct‑1 | developmental process (57.4%), negative regulation of cellular process (43.6%), negative regulation of biological process (44.7%), positive regulation of biological process (47.3%), response to organic substance (34.0%) | 192 |
| HNF1‑alpha | positive regulation of gene expression (27.4%), positive regulation of transcription from RNA polymerase II promoter (22.6%), positive regulation of transcription (26.3%), positive regulation of transcription, DNA‑dependent (23.7%), positive regulation of RNA metabolic process (23.7%) | 187 |
| SRF | positive regulation of cellular process (47.5%), developmental process (58.0%), positive regulation of macromolecule metabolic process (34.8%), positive regulation of metabolic process (35.9%), regulation of transcription from RNA polymerase II promoter (29.8%) | 185 |
