# Supplementary material for: A deficiency in SUMOylation activity disrupts multiple pathways leading to neural tube and heart defects in Xenopus embryos
Source: BMC Genomics. 2019 May 17;20:386. doi: 10.1186/s12864-019-5773-3 (PMC6525467; doi:10.1186/s12864-019-5773-3)
Supplement: Supplementary file 4 — Figure S3. Volcano plots of differentially expressed genes between Gam1 and control embryos. Fold change (log2) is plotted versus statistical significance at (A) early gastrula, (B) late gastrula, and (C) early neurula. Significance values of p < 0.05 (black line) indicates the cut off for accepted differential expression. (DOCX 22 kb) [file 12864_2019_5773_MOESM4_ESM.docx]

Table S4. SUMO Targets Sites and SUMO Interaction Motifs (SIM) in Top Transcription Factors from Network Building.

| Transcription Factor | SIM | SUMO Target Site | EG | LG | EN |
| --- | --- | --- | --- | --- | --- |
| C/EBPbeta‡ | + | – |  | * | * |
| c‑Jun‡ | + | + |  | * | * |
| NF‑Y | + | + |  | * | * |
| STAT1‡ | + | + |  | * | * |
| AP‑1‡ | + | + | * |  | * |
| C/EBPα‡ | + | + | * |  | * |
| CREB1‡ | + | + | * |  | * |
| STAT3V | + | + | * |  | * |
| Androgen receptor‡ | + | + | * | * | * |
| c‑Myc‡ | + | + | * | * | * |
| E2F1‡ | + | + | * | * | * |
| EGR1‡ | + | + | * | * | * |
| ESR1 (nuclear) | + | + | * | * | * |
| HIF1A‡ | + | + | * | * | * |
| HNF4α‡ | + | + | * | * | * |
| HSF1‡ | + | + | * | * | * |
| MYOD | + | + | * | * | * |
| Oct‑3/4‡ | + | + | * | * | * |
| p53‡ | + | + | * | * | * |
| RelA (p65 NF‑kB subunit)‡ | + | + | * | * | * |
| SP1‡ | + | + | * | * | * |
| SRF‡ | + | + | * | * | * |
| YY1‡ | + | + | * | * | * |
| ETS1‡ | + | + | * | * |  |
| AP‑4 | + | + |  |  | * |
| ATF‑2 | + | + |  |  | * |
| GATA‑1‡ | + | + |  |  | * |
| NF‑kB | + | + |  |  | * |
| p21 | + | + |  |  | * |
| p63‡ | + | + |  |  | * |
| SP3‡ | + | + |  |  | * |
| Bcl‑6 | + | + |  | * |  |
| c‑Myb‡ | + | + |  | * |  |
| E2F4 | + | + |  | * |  |
| GCRα‡ | + | + |  | * |  |
| HNF6 | ? | ? |  | * |  |
| Oct‑1 | + | + |  | * |  |
| SMAD3‡ | – | + |  | * |  |
| SOX4 | – | + |  | * |  |
| SREBP1 (nuclear) | + | + |  | * |  |
| TCF7L2 (TCF4)‡ | + | + |  | * |  |
| HNF1α | + | + | * |  |  |
| Sry | + | + | * |  |  |
| FKHR | ? | ? | * |  |  |
| NA–G | ? | ? | * |  |  |
| AP‑2‡ | – | + | * |  |  |
| ATF‑6α | + | + | * |  |  |
| ER81‡ | – | + | * |  |  |
| USF2 | + | + | * |  |  |
| FOXO3A | + | + | * |  |  |
| AP2A | – | + | * |  |  |

A question mark indicates that no *X. laevis* sequence for that transcription factor is present in the database. An asterisk indicates that the transcription factor is one of the top thirty which regulate the largest number of differentially expressed genes at that time point. ‡ is present next to transcription factors that were verified as SUMOylation targets through a literature search.
