# Supplementary figures and images for: A deficiency in SUMOylation activity disrupts multiple pathways leading to neural tube and heart defects in Xenopus embryos
Source: BMC Genomics. 2019 May 17;20:386. doi: 10.1186/s12864-019-5773-3 (PMC6525467; doi:10.1186/s12864-019-5773-3)

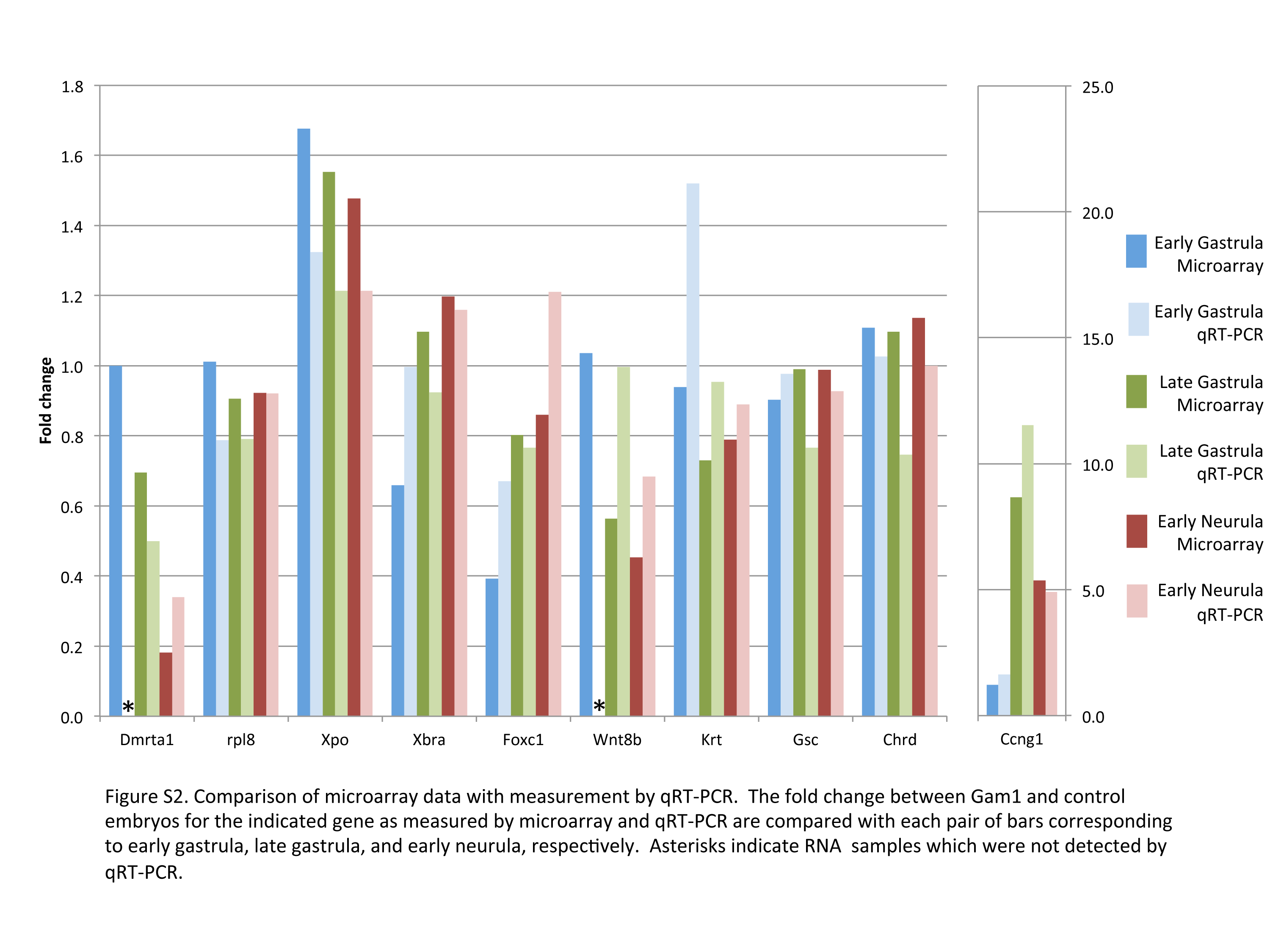

Supplement: Supplementary file 6 — Table S3. Biological Processes Associated with Transcription Factors Identified in Network Building. (TIF 21375 kb) [file 12864_2019_5773_MOESM6_ESM.tif]

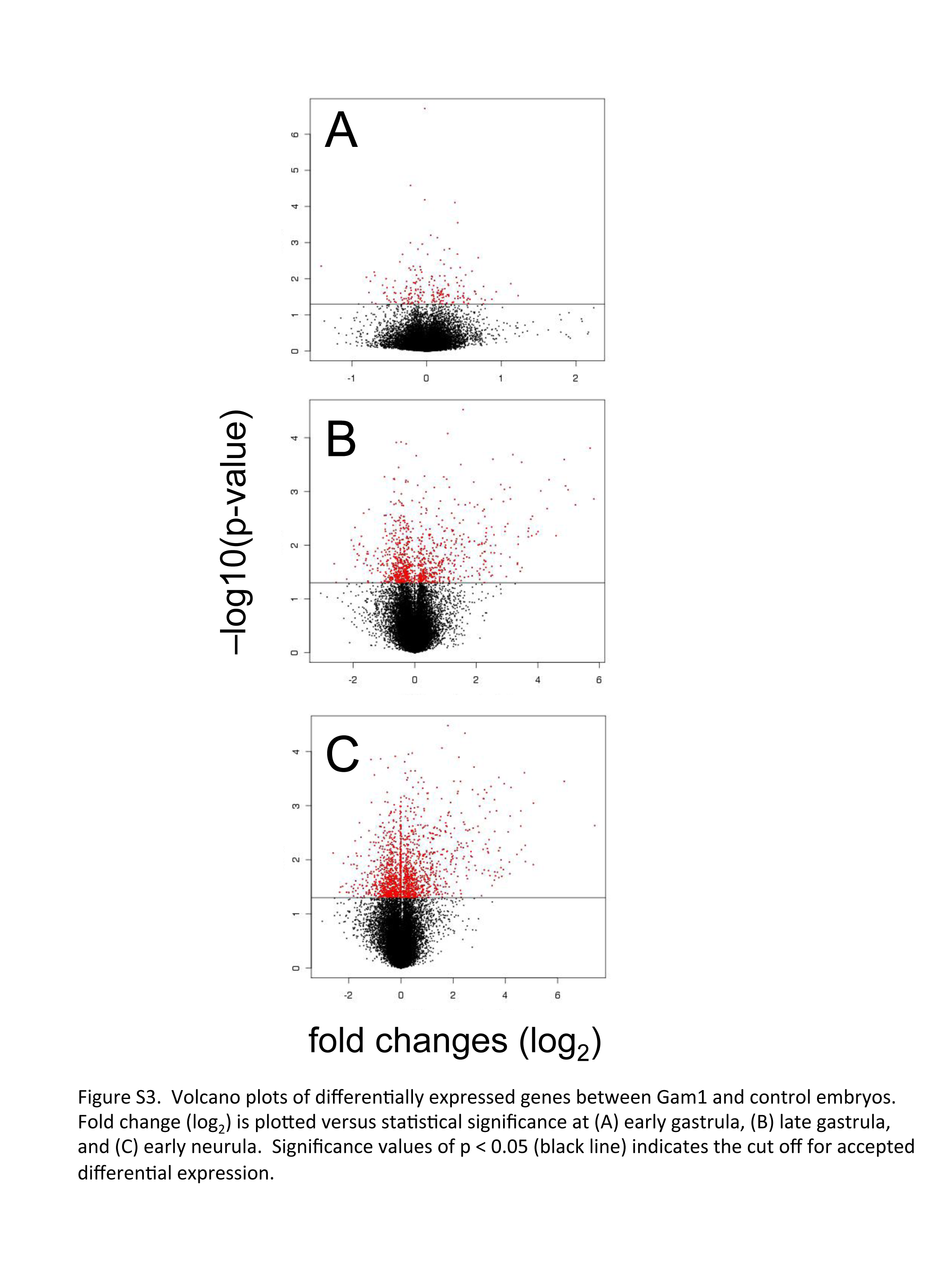

Supplement: Supplementary file 7 — Table S4. SUMO Targets Sites and SUMO Interaction Motifs (SIM) in Top Transcription Factors from Network Building. (TIF 21809 kb) [file 12864_2019_5773_MOESM7_ESM.tif]
